# Carbonic anhydrase and soluble adenylate cyclase regulation of cystic fibrosis cellular phenotypes

**DOI:** 10.1152/ajplung.00022.2021

**Published:** 2022-01-05

**Authors:** Kathleen Boyne, Deborah A. Corey, Pan Zhao, Binyu Lu, Walter F. Boron, Fraser J. Moss, Thomas J. Kelley

**Affiliations:** ^1^Department of Genetics and Genome Sciences, Case Western Reserve University, Cleveland, Ohio; ^2^Department of Physiology and Biophysics, Case Western Reserve University, Cleveland, Ohio; ^3^Department of Medicine and Department of Biochemistry, Case Western Reserve University, Cleveland, Ohio; ^4^Division of Pulmonary and Sleep Medicine, Department of Pediatrics, Ann & Robert H. Lurie Children’s Hospital of Chicago, Northwestern University, Feinberg School of Medicine, Chicago, Illinois

**Keywords:** bicarbonate, carbonic anhydrase, cystic fibrosis, microtubule

## Abstract

Several aspects of the cell biology of cystic fibrosis (CF) epithelial cells are altered including impaired lipid regulation, disrupted intracellular transport, and impaired microtubule regulation. It is unclear how the loss of cystic fibrosis transmembrane conductance regulator (CFTR) function leads to these differences. It is hypothesized that the loss of CFTR function leads to altered regulation of carbonic anhydrase (CA) activity resulting in cellular phenotypic changes. In this study, it is demonstrated that CA2 protein expression is reduced in CF model cells, primary mouse nasal epithelial (MNE) cells, excised MNE tissue, and primary human nasal epithelial cells (*P* < 0.05). This corresponds to a decrease in CA2 RNA expression measured by qPCR as well as an overall reduction in CA activity in primary CF MNEs. The addition of CFTR-inhibitor-172 to WT MNE cells for ≥24 h mimics the significantly lower protein expression of CA2 in CF cells. Treatment of CF cells with l-phenylalanine (L-Phe), an activator of CA activity, restores endosomal transport through an effect on microtubule regulation in a manner dependent on soluble adenylate cyclase (sAC). This effect can be blocked with the CA2-selective inhibitor dorzolamide. These data suggest that the loss of CFTR function leads to the decreased expression of CA2 resulting in the downstream cell signaling alterations observed in CF.

## INTRODUCTION

Cystic fibrosis (CF) is a progressive, autosomal recessive disease caused by mutations in the cystic fibrosis transmembrane conductance regulator (CFTR) gene. The CFTR protein is a cAMP-regulated Cl^–^ and HCO_3_^−^ channel located primarily at the apical membrane of epithelial cells, though its expression in other cell types has also been reported (1). Reduced or dysfunctional CFTR leads not only to decreased Cl^–^ transport and increased Na^+^ transport across epithelium, but also to alterations in several aspects of the cell biology of CF epithelial cells.

We have recently identified that microtubule regulation is altered in CF cells consisting of reduced tubulin acetylation and slower rates of reformation (2, 3). These alterations result in disrupted intracellular transport marked by reduced endosomal movement and accumulation in the perinuclear region (4). Microtubule dysfunction and intracellular transport can be restored in CF cells by inhibiting histone deacetylase 6 (HDAC6), a cytosolic deacetylase that regulates tubulin acetylation among other targets (5). We have also demonstrated that knocking out Hdac6 expression in a CF mouse model restores both linear growth and weight gain (6), as well as restores WT responses to airway bacterial challenge (7). Since evidence suggests that microtubule regulation is key to important CF phenotypes, we need to understand in more detail how the absence of CFTR function leads to microtubule changes.

One potential mechanism linking CFTR function to microtubule regulation is the cAMP pathway. We have previously shown that microtubule stability in CF cells is regulated in part by the exchange protein activated by cAMP (EPAC1) (8, 9). Specific stimulation of EPAC1 with the cAMP analog 8-cpt-2-*O*-methyl-cAMP (8-cpt-cAMPS) restored both microtubule dynamics and intracellular transport in CF cells. These data are consistent with the established role of EPAC1 as a plus-end microtubule-binding protein and contributor to microtubule elongation (10). Since CFTR is an important contributor to HCO_3_^−^ transport, HCO_3_^−^ regulation of cAMP production through soluble adenylate cyclase (sAC) is a potential avenue of microtubule control in CF cells (11, 12). In this study, we tested the hypothesis that aberrant regulation of carbonic anhydrases (CA), a zinc metalloenzyme responsible for catalyzing the reversible reaction of carbon dioxide and water to HCO_3_^−^ and H^+^, serves as a link between CFTR function and the alterations in the CF cellular phenotypes associated with microtubule regulation. Although previous studies have suggested that carbonic anhydrases play an essential role in CF, none of these investigations has been able to clearly link the loss of CFTR function directly to the alterations in the cell biology of CF epithelial cells (13, 14).

In this study, we demonstrate that carbonic anhydrase II (CA2) expression is reduced in CF model cells, primary mouse nasal epithelial (MNE) cells, and excised MNE tissue. These findings correspond to an overall reduction in CA activity in primary CF MNE cells. The strong correlation between CFTR function and CA2 expression is demonstrated by the addition of CFTR-inhibitor (inh172) to WT MNE cells that leads to significantly lower expression. The activation of CA2 with l-phenylalanine (L-Phe) restores intracellular transport, as measured by intracellular cholesterol movement, in a dose-dependent manner. This effect can be blocked with the CA2-selective inhibitor dorzolamide. L-Phe also restores microtubule polymerization in CF cells to more WT profiles. These data suggest that the loss of CFTR function leads to altered carbonic anhydrase regulation resulting in microtubule alterations and intracellular transport phenotypes in CF cells.

## METHODS

### Cells

9/HTEo-cells originally developed by Dr. Dieter Gruenert were human tracheal epithelial cells transformed by simian virus 40. They were grown at 37°C in a 95% O_2_-5% CO_2_ incubator on 10-cm diameter tissue culture dishes (Corning Inc., Corning, NY) in 10 mL of Dulbecco’s modified Eagle’s medium (DMEM; Gibco, Carlsbad, CA) supplemented with 10% FBS containing 2 mM l-glutamine, 0.04 mM HEPES, 1 unit/mL penicillin, and 1 µg/mL streptomycin. IB3 cells (CF), human bronchial epithelial cells isolated and immortalized from a patient with CF with ΔF508 mutation, and S9 cells (WT), IB3 cell mutation corrected with full-length WT CFTR, were a gift from Pamela L. Zeitlin (Johns Hopkins University, Baltimore, MD). They were grown at 37°C in a 95% O_2_-5% CO_2_ incubator on 10-cm diameter tissue culture dishes in LHC-8 basal medium (Gibco, Carlsbad, CA) supplemented with 5% FBS containing 1 unit/mL penicillin, and 1 µg/mL streptomycin. Primary mouse nasal epithelial (MNE) tissue was excised from WT (C57BL/6J, B6) and CF (F508del/F508del) mice. They were grown at 37°C in a 95% O_2_-5% CO_2_ incubator on T-75 flasks (Corning Inc., Corning, NY) in an F12 medium containing Y-27632, penicillin, and streptomycin. Primary human nasal epithelial cells were obtained from the Case Western Reserve University (CWRU) CF Center cell culture core facility.

### Mice

Wild-type (B6) and CF (F508del/F508del) mice were provided by the CF animal core facility (CWRU) between 10 and 12 wk of age. All mice were allowed unrestricted access to water and solid chow (Teklad 7904; Envigo). CF mice were allowed access to an osmotic laxative, PEG-3350 with electrolytes (Affordable Pharmaceuticals LLC) in water to avoid intestinal obstruction. All animals were maintained on a 12-h light, 12-h dark schedule at a mean ambient temperature of 22°C and were housed in standard polysulfone microisolator cages in ventilated units with corncob bedding. The Institutional Animal Care and Use Committee of CWRU approved all animal protocols.

### Western Blot Analysis of CA Protein Expression

CA1 (No. 13198-2-AP) antibodies were obtained from Proteintech (Rosemont, IL). CA2 (rabbit polyclonal) antibodies were obtained from Abcam No. 124687 KO validated (Cambridge, MA). Actin (No. H-6) antibody was obtained from Santa Cruz Biotechnology (Santa Cruz, CA). Cells were grown to 95% confluency in 35-mm dishes and lysed with lysis buffer (50 mM Tris pH 7.5, 1% Triton X-100, 50 mM NaF, 200 μM Na_3_VO_4_, and 10 μg/mL pepstatin and leupeptin) for 20 min at 4°C. Lysates were centrifuged at 10,000 rpm for 5 min. Proteins were separated using SDS-PAGE containing 20–40 μg protein on 10% polyacrylamide gels. Samples were transferred to an Immobilon-P membrane (Millipore, Bedford, MA) at 15 V for 30 min. Membranes were blocked in 10% nonfat dehydrated milk in PBS with 0.1% Tween-20 (PBS-T) for 1 h at room temperature and then incubated with primary antibodies diluted in 10% nonfat dehydrated milk in PBS-T overnight at 4°C. Membranes were washed three times for 10 min each with PBS-T, incubated with the respective secondary antibodies conjugated to horseradish peroxidase (1:3,000 dilution) in PBS-T, washed again with PBS-T, and visualized using SuperSignal chemiluminescent substrate (Pierce, Rockford, IL) and the Chemidoc Imaging System (Bio-Rad, Hercules, CA). Quantification of protein expression was performed with Quality One software (Bio-Rad).

### RNA qPCR of CA Expression

MNE cells were grown in 35-mm dishes to 95% confluency and rinsed with 1× PBS. RNA was isolated with TRIzol (500 µL/well). After 5 min of incubation at room temperature, 100 µL chloroform was added per 500 µL of TRIzol. Following vigorous shaking and a 2–3-min incubation period, samples were spun at 12,000 rpm for 15 min at 4°C. The aqueous layer was removed and 250 µL of isopropanol was added. Samples were mixed, incubated for 10 min at room temperature, and then spun at 12,000 rpm for 10 min at 4°C. Supernatant was discarded and the pellet was washed in 75% ethanol. Samples were spun at 7,500 rpm for 5 min at 4°C then supernatant removed, and pellet air-dried for 5 min. Pellets were resuspended in RNAse-free water and quantified on the NanoDrop. Then cDNA was synthesized using a qScript cDNA synthesis kit (Quantabio). TaqMan gene expression assay (Applied Biosystems No. 4369016) was performed and experiment was run using StepOne Real-Time PCR System. Results were analyzed using the Comparative CT method.

### Filipin Cholesterol Staining

Cells were grown on collagen-coated coverslips to 75%–90% confluency. They were treated for 24 h with l-phenylalanine (100–500 µM) with or without Dorzolamide (15 µM), a CA2-selective inhibitor, and/or KH7 (25–50 µM) or LRE1 (25 µM), soluble adenylate cyclase inhibitors. Cells were rinsed with PBS then fixed with 2%–4% paraformaldehyde for 15–30 min at room temperature. Cells were rinsed again then incubated with filipin for 1 h on the shaker in the dark. Cells were visualized in the ultraviolet range with a ×40 oil objective on a wide-field Leica DM6000 upright microscope with Improvision’s Volocity software. The number of cells having or not having perinuclear cholesterol accumulation was counted, and quantification was determined by the ratio of cells with accumulation to total cells. Over 30 images were quantified for each condition.

### Rab7 Late Endosome Staining

Rab7 (rabbit) antibodies were obtained from Abcam (Cambridge, MA). Cells were grown on collagen-coated coverslips to 75%–80% confluency. Cells were rinsed and fixed with cold methanol for 20 min at −20°C. Cells were blocked in 5%–10% goat serum for 30 min, incubated in 5%–10% goat serum with Rab7 antibodies (1:150) for 1 h, and incubated with Texas Red goat anti-rabbit IgG antibodies in 5%–10% goat serum in the dark. Cells were rinsed and stained with 1 µg/mL of 4',6-diamidino-2-phenylindole (DAPI) in PBS for 5 min in the dark. Cells were mounted with SlowFade (Invitrogen) on slides, and they were visualized in the appropriate range using a Leica DM6000 upright microscope (×40 oil objective) with Improvision’s Volocity software. Data were analyzed using ImageJ software. Perimeters were drawn around each nucleus and partial perimeters, highlighting the portion of the nucleus with endosomal accumulation, were drawn on top of these. Measurements of the length of these segments were generated on the software and used to calculate the ratio of the covered nuclear circumference. A *t* test was performed to compare the ratios of perinuclear accumulation between treated and mock-treated cells. Every cell with a nucleus fully visible in the frame of the image that did not appear to be dividing was measured by this method and included in the analysis.

### Microtubule Elongation

Cells were grown on collagen-coated coverslips to 75%–90% confluency. Cells were removed from the 37°C 5% CO_2_ incubator and placed on ice for 45–60 min. After this depolymerization period, prewarmed 37°C media (with vehicle or drug) was added at designated time points (0–20 min). At the end of the time course, cells were rinsed with PBS and fixed at the indicated time points. Cells were immunostained according to Rab7 staining protocol.

Mouse and S9/IB3 cells were fixed and permeabilized with methanol for 20 min at −20°C then rinsed with PBS and blocked with 5% goat serum in PBS for 30 min at room temperature. HNE cells were fixed with 2% paraformaldehyde and permeabilized with 0.1% NP-40 then blocked with 5% goat serum according to Rab7 staining protocol. Cells were incubated for 1 h on the shaker with α-tubulin (rabbit) antibody from Abcam (Cambridge, MA) diluted 1:200 in blocking solution. After rinsing with PBS, the secondary antibody (anti-rabbit IgG Fab2 AlexaFluor 594 Cell Signaling) diluted 1:5,000 in blocking solution was added. Cells were incubated for 1 h at room temperature on the shaker in the dark then rinsed and mounted on slides. Cells were visualized in the appropriate range using a Leica DM6000 upright microscope (×40 oil objective) with Improvision’s Volocity software. For each time point, 5–10 representative fields were captured, yielding 40–100 cells. Each cell was scored as having or not having an aster present and quantification was determined by the ratio of cells with an aster, indicating microtubule formation, to total cells at set time points.

### Effect of CA2 Knockdown by siRNA Studies

CA2 expression knockdown in IB3 cells was accomplished using CA2 esiRNA (Sigma-Aldrich, Catalog No. EHU155141) transfected with Fugene HD (Promega) with pMAX-GFP (Lonza Bioscience, Catalog No. V4XC-9064) as a transfection marker. Briefly, 100 ng each empty pMAX-GFP and CA2 siRNA were added to 250 mL Optimem along with 8 mL Fugene HD (for a 1:2 DNA:Fugene HD ratio). This transfection mix was incubated at RT for 20 min. During incubation, plating media was removed and 250 mL of fresh IB3 media was added to each well. After incubation, 250 mL of this transfection mix was added to each well. Cells were incubated overnight at 37°C^,^ 5% CO_2_. Cells are treated with or without 500 µM L-Phe for 24 h and then fixed and stained for Rab7. Parallel experiments were done for the determination of CA2 knockdown efficiency. Twenty-four hours posttransfection, cells were sorted for GFP fluorescing cells using the nanocellect cell sorter (Nanocellect, San Diego, CA). Briefly, nontransfected IB3 cells were rinsed with filtered PBS and lifted off the plate with filtered trypsin. Cells were centrifuged at 900 rpm, 5 min, and counted using trypan blue and the countess counter. Cells were resuspended at a concentration of 1 million cells per milliliter in IB3 media + 10% trypsin. This solution was passed through a 0.40 μm mesh strainer to ensure single-cell suspension. This cell suspension was used in the WOLF sorter to gate live cells. Gated, live cells were collected to be used as a nontransfection control. Once cells were gated to select for live cells only, transfected IB3 cells which were prepared in the same manner were passed through the sorter and gated so that only GFP fluorescing cells were collected. Total RNA was isolated from the sorted cells using Qiagen’s MicroRNA columns according to the manufacturer’s protocol. Fifty nanograms of total RNA were reverse transcribed into cDNA using qScript cDNA synthesis kit (QuantaBio). cDNA from the above reaction was then used to quantify CA2 gene expression using TaqMan primers (Thermo Fisher).

### Ussing Chamber Analysis

Short-circuit current measurements were performed in WT human bronchial epithelial (HBE) cells. HBE cells were obtained from CF cell culture core facility and cultured as described with HNE cells in *Cells*. After 3 wk, the establishment of electrically tight cultures was determined by Ussing chamber measurements at which time electrophysiological analysis was completed. For testing treatments, epithelial cells were treated with either vehicle (water) or L-Phe (500 µM) for 24 h. The epithelial monolayers were then bathed with symmetrical Krebs bicarbonate Ringer’s solution and maintained under short-circuit conditions. Amiloride, forskolin, and CFTR inhibitor inh172 were added sequentially to inhibit Na^+^ absorption, stimulate CFTR-dependent Cl^–^ secretion and inhibit CFTR activity to determine how much current was due to CFTR function, respectively.

### pH Physiology: Solutions

The standard HEPES-buffered solution (HBS) contained (in mM): 113.6 NaCl, 5 KCl, 2 NaH_2_PO_4_, 1 CaCl_2_, 1.2 MgSO_4_, 32.5 HEPES, and 10.5 glucose titrated to pH 7.4 at 37°C with NaOH. For the 5% CO_2_/22 mM HCO_3_^−^ solution, the non-CO_2_/HCO_3_^–^ components were dissolved at the following concentrations (in mM): 91.6 NaCl, 5 KCl, 2 NaH_2_PO_4_, 1 CaCl_2_, 1.2 MgSO_4_, 32.5 HEPES, and 10.5 glucose, then titrated to pH 7.4 at 37°C with NaOH. NaHCO_3_ was then added for a final concentration of 22 mM and finally, the solution was equilibrated with CO_2_ for 30−40 min using a computerized gas-mixing system (Series 4000, Environics, Tolland, CT). For 100 µM acetazolamide (ACZ; Sigma-Aldrich, St. Louis, MO, A6011) solutions, we made a 50 mM stock in 0.05 N NaOH and added 2 mL/L of this 50 mM stock to the HBS or CO_2_/HCO_3_^−^ solutions. In the 100 µM ACZ CO_2_/HCO_3_^−^ solution, the ACZ stock was added together with the non-CO_2_/HCO_3_^–^ components and the solution pH was set to 7.4 at 37°C, before the addition of the NaHCO_3_ equilibration with CO_2_. We measured solution osmolalities using a vapor-pressure osmometer (5520, Wescor Inc, Logan, UT), and adjusted the osmolality to 300 ± 5 mosmol/kgH_2_O.

### pH Physiology: Fluorescence Recordings

pH_i_ recordings were performed using a previously described imaging setup (15–17). Briefly, a coverslip with cells attached was removed from the incubator and fixed to our perfusion chamber, forming its floor. To the assembled chamber, we added 200 µL of HBS containing 10 µM of the pH-sensitive dye (2',7'-bis-2-carboxyethyl)-5(and-6) carboxyfluorescein acetoxymethyl ester (BCECF-AM; B1170, Thermo Fisher Scientific, Waltham, MA) and incubated for 10 min at room temperature. We then mounted the chamber on the automated stage of an Olympus IX-81 microscope equipped with epi-fluorescence imaging. We immediately commenced the flow of HBS at 37°C, alternately exciting the BCECF at 440 nm and 490 nm, while recording at emission wavelengths >530 nm, to record changes in pH_i_. The exposure time for each excitation wavelength was 100 ms, separated by ∼20 ms. We acquired a 440-nm (*I*_440_) and a 490-nm (*I*_490_) intensity data pair every 5 s. We delivered the solutions to the experimental chamber at 3 mL·min^−1^ using syringe pumps (model 33, Harvard Apparatus, Holliston, MA). We selected between solutions using a computerized valve system and maintained solution temperature at 37°C by means of a water-jacket system placed between the valves and the chamber. Slidebook 6.0.14 software (Intelligent Imaging Innovation, Denver, CO) provided data acquisition.

For each cell analyzed, we selected an area of interest (AOI) by using the outline tool in Slidebook to encompass the cell body. We calculated the rate constant describing the rate of change of *I*_440_ (−*k*_440_) continuously throughout our experiments as an index of membrane integrity. We regarded the cells as healthy if the absolute value of −*k*_440_ is less than 5% min^−1^ (18), and only included healthy cells in the analyses.

Experiments began by flowing HBS over BCECF-loaded MNE cells for at least 5 min until the baseline pH_i_ stabilized. We then switched the perfusing solution to 5% CO_2_/22 mM HCO_3_^−^ for 4 min, before returning the bath solution to HBS for 5 min. We then switched the bath solution to HBS + 100 µM ACZ for additional 5 min before switching to 5% CO_2_/22 mM HCO_3_^−^ + 100 µM ACZ for 4 min then returning to HBS for 5 min.

We computed the pH_i_ values by using the high-K^+^-nigericin technique (19). At the end of each recording (after the final HBS pulse), we applied Na-free, high-K^+^ solutions containing 10 µM nigericin (N1495, Thermo Fisher Scientific) and 135 mM K^+^, buffered at five pH values of 5.8, 6.4, 7.0, 7.6, and 8.5 for each coverslip. The *I*_490_/*I*_440_ ratio data for each cell is described by a pH titration curve:

(*1*) $$\frac{I_{490}}{I_{440}}=a+b\frac{10^{\left(pH-pK\right)}}{1+10^{\left(pH-pK\right)}}$$

We used *Eq. 1* to calculate pH_i_ from *I*_490_/*I*_440_ ratios and the fitted values for p*K* and *b* for each cell. The initial rate of CO_2_-induced pH_i_ acidification (d(pH_i_/dt)_max_) was analyzed by fitting the pH_i_ versus time record with a straight line. The mean d(pH_i_/dt)_max_ data were analyzed by one-way analysis of variance (ANOVA) and we controlled for type I errors across multiple means comparisons with a Holm–Bonferroni correction (18), setting the familywise error rate (FWER) to α = 0.05. Briefly, we order the unadjusted *P* values for all η comparisons in each data set from lowest to highest. For the first test, we compare the lowest unadjusted *P* value with the first adjusted α value, α/η. If the null hypothesis is rejected, then we compare the second-lowest *P* value to the second adjusted α value, α/(η–1), and so on, until if at any point the unadjusted *P* value is ≥ the adjusted α, the null hypothesis is accepted and all subsequent hypotheses in the test group are considered null.

### Stopped-Flow Assay for Quantitating Carbonic Anhydrase Activity

Two days before the assay, 2 × 10^6^ WT or CF (F508del/F508del) MNE cells were plated into T75 flasks. Twenty-four hours before commencing the assay, the media was changed to exclude Y-27632. On the day of the assay, media was aspirated from each flask, and the cells were washed with 1 mL of sterile PBS. The PBS was aspirated and 1 mL of enzyme-free cell dissociation buffer (Gibco 13151-014) was added to each flask and incubated at room temperature for 2 min. Cells were then scraped from the base of each flask, and pelleted in a centrifuge for 10 min at 2,000 *g*. The dissociation solution was aspirated and the cell pellet resuspended with 100 µL of washing solution [containing (in mM): 92.5 NaCl, 46.98 Na_2_HPO_4_, 11.02 NaH_2_PO_4_, and 0.01 CaCl_2_, and cOmplete mini, EDTA-free protease inhibitor cocktail (one tablet/10 mL, Roche Applied Biosciences No. 11836170001, Indianapolis, IN) adjusted at room temperature to pH to 7.4 using NaOH and final osmolality ∼300 mosmol/kgH_2_O]. 1 cell-pellet volume (∼100 µL) 0.5 mm zirconium oxide beads (ZROB05, Next Advance Inc., Troy, NY) were then added to each tube and the samples homogenized at 4°C using a Bullet Blender 24 Gold (Next Advance Inc.) set at speed 8 and time 3. The protein concentration of each lysate sample was determined using Quick Start Bradford Protein Assay (Bio-Rad 5000205, Hercules, CA). An appropriate volume of each cell lysate was then added to OOE buffer A [containing (in mM):140 NaCl, 3 KCl, 2 CaCl_2_, and 16 HEPES adjusted to pH 7.03 and final osmolality ∼295 mosmol/kgH_2_O at 10°C] for a final total protein concentration of 1 mg/mL in a 2 mL volume. The CA assay was performed as previously described (20), mixing protein/OOE buffer A solution equally with OOE buffer B (containing 2 µM pyranine and (in mM):116 NaCl, 3 KCl, 44 NaHCO_3_, ∼1% CO_2_ adjusted to pH 8.41, and final osmolality ∼300 mosmol/kgH_2_O at 10°C), The final total protein concentration in the stopped-flow cell was 0.5 µg/mL. We fitted the pH time course with *Eq. 2*:

(*2*) $$\text{pH}(t)=A-Be^{-(k_{\Delta pH})t}$$

where *t* is time, *A* is the final (equilibrated) value of pH, *B* is the pH range, and *k*_ΔpH_ is the rate constant of the pH relaxation. We obtained *A*, *B*, and *k*_ΔpH_ using a nonlinear least-squares method.

## RESULTS

### Carbonic Anhydrase Activity in Cystic Fibrosis Cells

Because the HCO_3_^−^ sensor sAC is a key regulator of CF-cellular phenotypes, a key factor becomes the regulation of intracellular [HCO_3_^−^] ([HCO_3_^−^]_i_) in CF airway cells. CFTR is an HCO_3_^−^ transporter and several reports have shown disrupted HCO_3_^−^ regulation in CF cells (21–24). Another factor in [HCO_3_^−^]_i_ regulation is the activity of numerous carbonic anhydrases. Carbonic anhydrases (CAs) are ubiquitous metalloenzymes that serve important roles regulating numerous physiological processes, including transepithelial HCO_3_^−^ (25) and fluid transport (26), CO_2_ carriage by red blood cells (27), the speed of intracellular pH (pH_i_) transients (28), and transient extracellular pH changes in the brain (29). The human genome contains 16 human CAs, 13 of which are known to be active (30) in catalyzing the reaction CO_2_ + H_2_O·HCO_3_^−^ + H^+^, eight are cytosolic; and the remainder are mitochondrial, GPI-anchored, or transmembrane in location (31, 32).

**Figure 1.**
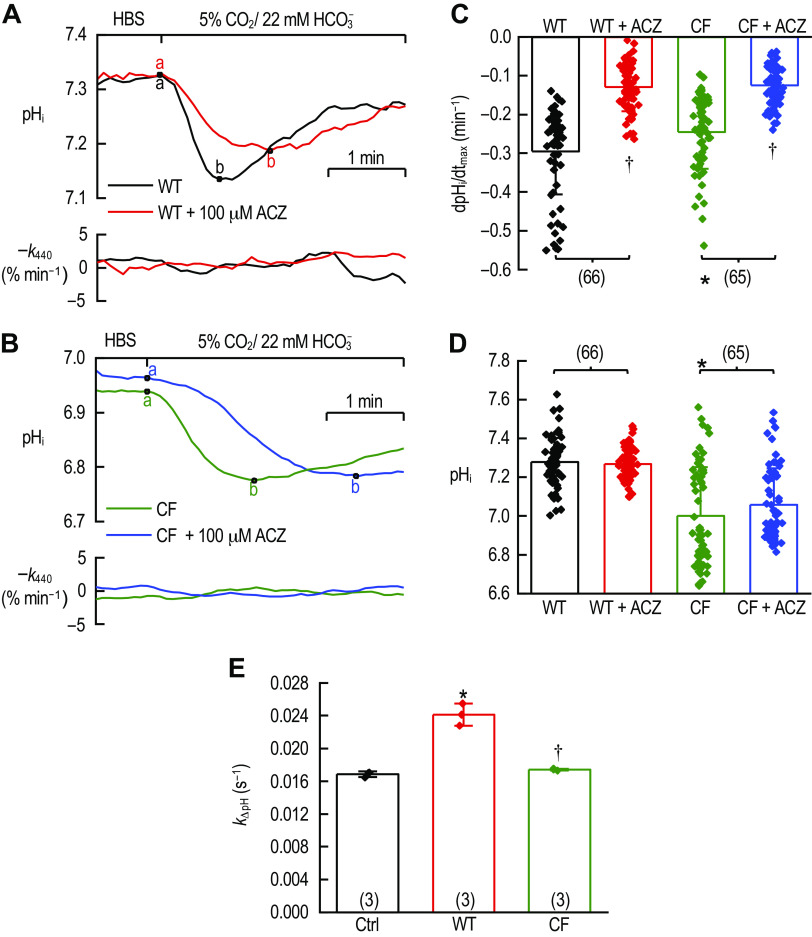
CA activity in WT and CF MNE cells. *A* (*top)*: representative pH_i_ trace from a WT MNE cell before (black) and after (red) 5 min perfusion with 100 µM ACZ. The recording commences with the cell being perfused by CO_2_/HCO_3_^−^-free HBS (pH 7.4). The baseline pH_i_ is allowed to stabilize until point *a*, where we switch the perfusing solution to 5% CO_2_/22 mM HCO_3_^−^ (pH 7.4). CO_2_ enters the cell and produces HCO_3_^−^ and H^+^ lowering pH_i_ until it nadirs at point *b*. *bottom*: the rate constant describing the loss of the *I*_440_ signal (–*k*_440_) from the same cell displayed in the pH_i_ recordings. Cells are considered healthy if the absolute value of –*k*_440_ is ≤5% min^−1^. *B*: representative pH_i_ (*top*) and –*k*_440_ (*bottom*) traces from a CF MNE cell before (blue) and after (green) 5 min perfusion with 100 µM ACZ. Labels are as otherwise described for *A*. *C*: bars represent the mean (± SD) (dpH_i_/dt)_max_ during each CO_2_-mediated intracellular acidification (points *a* → *b*, *A* and *B*) from WT MNE cells before (black) and after (red) 100 µM ACZ perfusion, and CF MNE cells before (blue) and after (green) 100 µM ACZ. *D*: bars represent the mean (± SD) initial pH_i_ recorded at point *a* in *A* and *B* for WT MNE cells before (black) and after (red) 100 µM ACZ perfusion, and from CF MNE cells before (blue) and after (green) 100 µM ACZ. For both *C* and *D*, each replicate is displayed as a symbol overlaid on the bar, with the total number of replicates displayed in parentheses above the bars. *Mean value is significantly different from WT. †Mean value is significantly different from the pre-ACZ value for the same cell type. *E*: CA activity from WT and CF MNE whole cell lysates measured by a stopped-flow assay based on mixing of two solutions to create an OOE CO_2_/HCO_3_^−^ state. Bars represent the mean (± SD) *k*_ΔpH_ from 0.5 mg/mL cell lysate. *Mean value is significantly different from the protein-free control. †Mean value is significantly different from WT. Each replicate is displayed as a symbol overlaid on the bar, with the total number of replicates displayed in parentheses. For *C–E*, statistical significance was determined by analysis of variance with a Holm–Bonferroni analysis method to control for type I errors across multiple comparisons. ACZ, acetazolamide; CA2, carbonic anhydrase II; CF, cystic fibrosis; HBS, HEPES-buffered solution; MNE, mouse nasal epithelial; OOE, out-of-equilibrium; WT, wild-type.

To examine CA activity present in WT and CF MNE cells, we measured the maximal rate of CO_2_-induced pH_i_ change [(dpH_i_/dt)_max_], during two 4-min 5% CO_2_/22 mM HCO_3_^−^ pulses separated by 5 min in HBS and an additional 5 min in HBS + 100 µM ACZ (a CA inhibitor). Figure 1*A* shows a representative pH_i_ trace from a WT MNE cell before (black) and after (red) treatment with 100 µM ACZ. Following the switch of the bath solution from HBS to 5% CO_2_/22 mM HCO_3_^−^ (point a), pH_i_ falls rapidly as CO_2_ enters the cell and produces HCO_3_^−^ and H^+^, the latter of which is sensed by the BCECF dye in the cytoplasm. The pH_i_ continues to acidify until it reaches its nadir (point b) when the net acid load and acid extrusion rates are equal. After point b, the cell’s acid extruding mechanisms begin to dominate as the CO_2_ nears or reaches equilibrium across the plasma membrane, and we observe a net alkalinization of the pH_i_. In the hypothetical scenario that the cell does not express any acid-extruding proteins (e.g., CFTR, Na-H exchangers, or HCO_3_^−^ transporters from the *scl4* gene family), the pH_i_ would remain steady around its acidic nadir until such time that the extracellular solution is switched to one lacking dissolved CO_2_. CA activity will speed the rate at which pH_i_ falls during CO_2_-mediated intracellular acidification (points *a* → *b*), but not change the final pH_i_ (28, 33, 34).

Comparing the mean (dpH_i_/dt)_max_ from WT MNE cells before (Fig. 1*A*, black trace), and after 5 min perfusion of 100 µM ACZ (Fig. 1*A*, red trace), we find that the mean (dpH_i_/dt)_max_ post-ACZ is significantly reduced to 48.0 ± 3.3% (errors are SE) of the value measured in the same cells, pre-ACZ treatment (Fig. 1*C*, black vs. red points, *P* = 8.11 × 10^−25^, α = 0.01, *n* = 66). Similarly, when we compare the mean (dpH_i_/dt)_max_ from CF MNE cells before (Fig. 1*B*, green trace), and after 5 min perfusion of 100 µM ACZ (Fig. 1*B*, blue trace), we observe that mean (dpH_i_/dt)_max_ post-ACZ perfusion is reduced to 55.2 ± 3.4% compared with that measured from the same cells pre-ACZ treatment (Fig. 1*C* green vs. blue points, *P* = 6.34 × 10^−15^, α = 0.0125, *n* = 65). Therefore, both WT and CF cells exhibit ACZ-sensitive CA activity. In the presence of ACZ, (dpH_i_/dt)_max_ from WT cells (WT + ACZ) and CF cells (CF + ACZ) are not significantly different from one another (Fig. 1*C*, red vs. blue bars, *P* = 0.725, α = 0.05, *n* = 66 vs. 65). However, in the absence of ACZ, the mean (dpH_i_/dt)_max_ from CF cells was 17% less than that from WT cells (Fig. 1*C*, black vs. green bars, *P* = 6.81 × 10^−04^, α = 0.025, *n* = 66 vs. 65), consistent with the hypothesis that CF cells have reduced CA activity. However, we observed that the initial pH_i_ (point a, Fig. 1, *A*, *B*, and *D*), before the switch to 5% CO_2_/22 mM HCO_3_^−^ is significantly and substantially lower (by ∼0.3 pH units) in CF cells versus WT cells. Regardless of any possible change in CA activity in the CF cells, the lower initial pH_i_, the closer that pH_i_ is to p*K*_a_, the less the tendency of entering CO_2_ to undergo the reactions CO_2_ + H_2_O·H_2_CO_3_ → HCO_3_^−^ + H^+^, and thus the slower the intracellular acidification [i.e., the smaller the magnitude of (dpH_i_/dt)_max_] (35). Based on the initial pH_i_ values, we estimate that the lower pH_i_ of CF cells would have reduced the magnitude of (dpH_i_/dt)_max_ by ∼5%, which is substantially less than the observed reduction in (dpH_i_/dt)_max_.

To determine whether the difference in (dpH_i_/dt)_max_ was attributable to reduced CA activity or the more acidic initial pH_i_ in CF cells compared with the WT cells, we directly measured the total CA activity in whole cell lysates using a stopped-flow assay based on the mixing of two solutions to create an out-of-equilibrium (OOE) CO_2_/HCO_3_^−^ state (20). In this assay, the pH-relaxation rate constant (*k*_ΔpH_) rises linearly from the baseline with increases in sample CA activity. Figure 1*E* reports that the baseline *k*_ΔpH_ in the OOE sample buffer (Ctrl, black bar) was 0.0169 ± 0.0002 s^−1^ (error is SE) representing the rate constant of the uncatalyzed reactions that occur during the pH relaxation under conditions of the assay at 10°C. Lysate from WT MNE cells exhibited CA activity (red bar, *P* = 3.4 × 10^−5^, *n* = 3) significantly greater than the Ctrl, but CA activity in the CF cells was substantially reduced compared with WT (green bar, *P* = 5.5 × 10^−5^, *n* = 3), and not significantly greater than measured in Ctrl samples (*P* = 0.4, *n* = 3). These results indicate that the reduced (dpH_i_/dt)_max_ measured from CF cells compared with WT (Fig. 1*D*) was mainly due to a significant reduction in the intracellular CA activity.

### Expression of Carbonic Anhydrases in Cystic Fibrosis Cells

**Figure 2.**
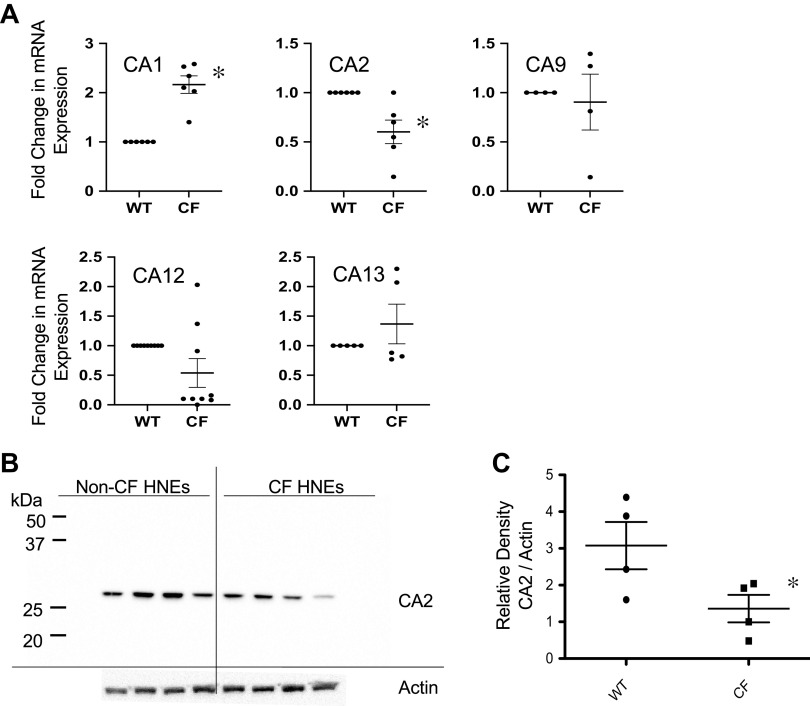
Expression of CA2 in CF cells. *A*: RNA expression of CA1, CA2, CA9, CA12, and CA13 in WT and CF MNE. CA1 expression is significantly higher in CF compared with WT (*n* = 6 for each, *P* < 0.001 by *t* test). CA2 expression is significantly lower in CF compared with WT (*n* = 6 for each, *P* < 0.001 by *t* test). CA12 expression trends toward lower but did not reach significance. CA4 and CA8 expression was also measured but not detected. *B*: protein expression of CA2 in primary human nasal epithelial (HNE) cells from four separate F508del homozygous subjects. Actin expression used as a control. *C*: quantification of CA2 expression reported as relative density (CA2/actin). Significance determined by *t* test; *P = 0.03, *n* = 4. CA2, carbonic anhydrase II; CF, cystic fibrosis; MNE, mouse nasal epithelial; WT, wild-type.

Carbonic anhydrases are relevant to CF as demonstrated by CA12 mutations in individuals presenting with a CF-like phenotype, including elevated sweat Cl^–^ levels and lung disease (36). To begin assessing CA expression levels in our model system, RNA expression of CA in primary cultured MNE cells was investigated using qPCR. RNA-Seq data from WT MNE cells indicated that CA1, CA2, CA4, CA8, CA9, CA12, and CA13 were expressed (data not shown). Expression of these carbonic anhydrases was compared in WT and CF MNE by qPCR. CA1 showed a significant increase and CA2 a significant decrease in expression in CF (F508del/F508del) MNE compared with WT in this assay (Fig. 2*A*). CA4 and CA8 were not detected. CA12 showed a strong trend toward reduction, though not statistically significant. CA2 expression is altered in response to knockout of another HCO_3_^−^ transporter, anion exchange protein (AE2), and maybe more associated with sAC function (37). Given the cytosolic location of CA2 with a consistent CF phenotype of decreased expression in our study, this was the focus for investigations of CA’s impact on the disrupted cellular signaling cascade in CF though clearly other CAs may be important to overall CF cell regulation as well.

**Figure 3.**
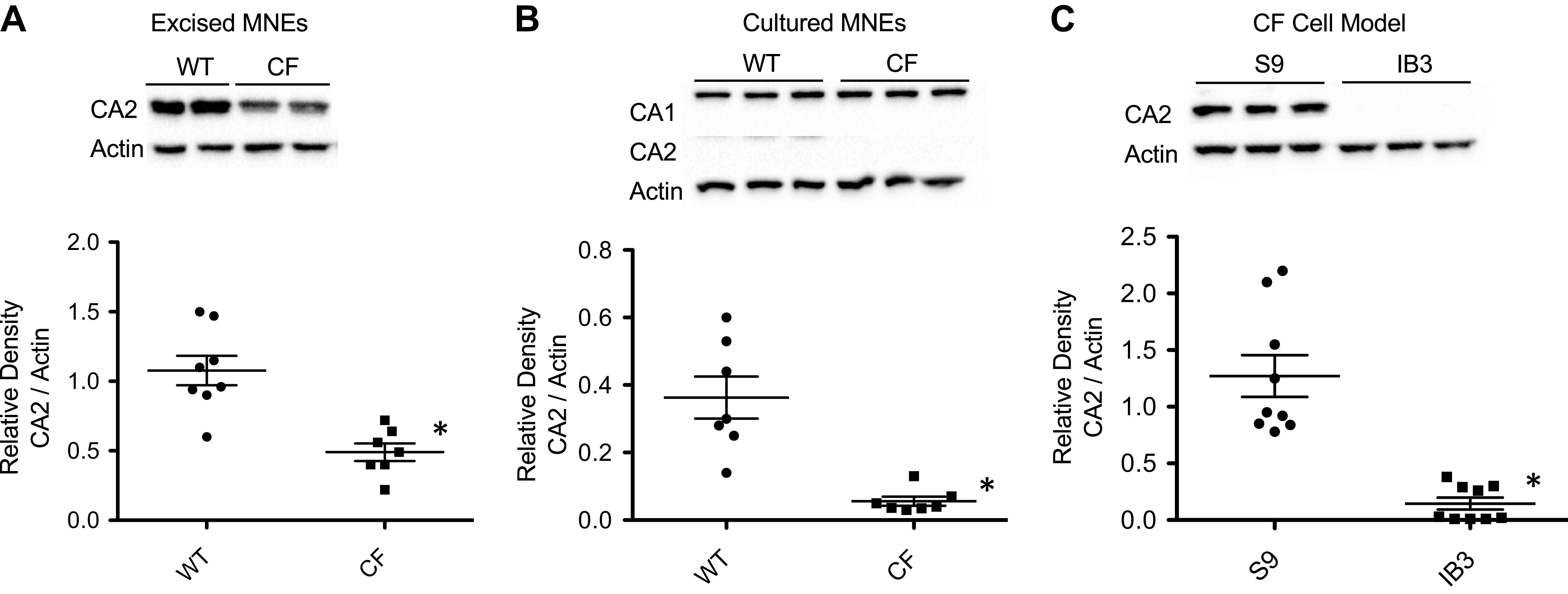
CA2 protein expression is reduced in CF cell model, cultured primary mouse nasal epithelial (MNE) cells, and excised MNE tissue. *A*: CA2 expression is decreased in CF (F508del) primary excised MNE tissue compared with WT controls (*n* = 10). *B*: CA2 expression is similarly decreased in cultured CF primary MNE cells (*n* = 4), whereas CA1 expression is the same (*n* = 3). *C*: cultured CF cell model IB3 cells demonstrate a near complete lack of CA2 expression compared with corrected control cells. (*n* = 3, in triplicate). Significance determined by *t* test; *P ≤ 0.001. CA2, carbonic anhydrase II; CF, cystic fibrosis; WT, wild-type.

In addition, Western blot was used to evaluate CA2 expression at the protein level. There was significantly decreased expression of CA2 at the protein level in primary CF compared with non-CF HNE cells (*n* = 4, *P* < 0.05) (Fig. 2, *B* and *C*). Figure 3 illustrates similarly significantly decreased expression of CA2 in CF primary excised MNE tissue (*n* = 7, *P* < 0.0001) (Fig. 3*A*), cultured CF MNE cells (*n* = 9, *P* < 0.0001) (Fig. 3*B*), and CF model cells, demonstrating almost complete lack of CA2 expression in IB3 cells (*n* = 3 in triplicate, *P* = 0.0001) (Fig. 3*C*). CA1 protein expression was also assessed in CF- versus non-CF-cultured MNE cells for comparison to ensure this was not a global relationship with all CAs (*n* = 3). Expression of CA1 was not significantly different in CF cells (Fig. 3*B*). These results of reduced CA2 expression are consistent with previously published data from CF mouse intestine (13), but not with Walker et al. (38), who found no change in CA2 expression in Cftr KO crypt epithelial cells.

### Carbonic Anhydrase II Expression Can Be Directly Reduced by Inhibition of CFTR

**Figure 4.**
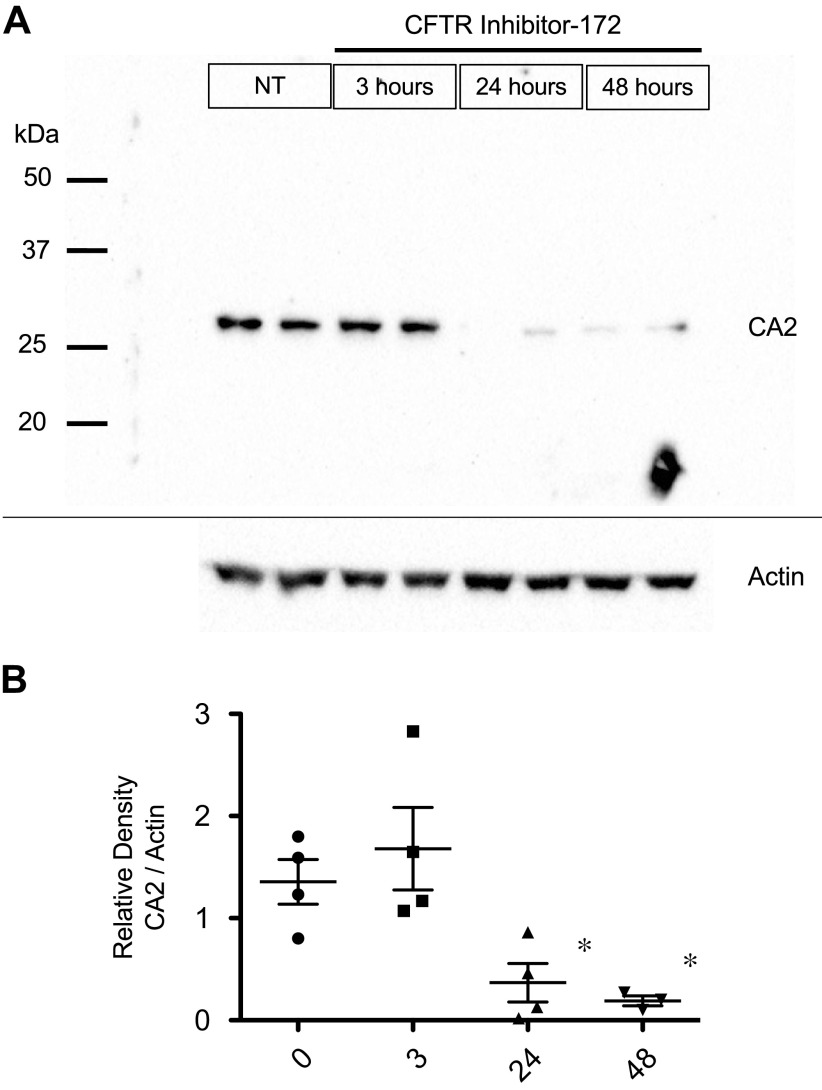
Reduction in CA2 expression can be induced by treatment of wild-type (WT) primary mouse nasal epithelial (MNE) cells with CFTR inhibitor-172. WT MNE cells were treated for 0 (*n* = 4), 3 (*n* = 4), 24 (*n* = 4), and 48 (*n* = 3) h with 100 µM inh172. *A*: representative gel showing CA2 expression in response to treatment with actin as a loading control. *B*: quantification of CA2 expression reported as relative density (CA2/actin). Significance determined by ANOVA with Newman–Keuls multiple comparison test; *P < 0.05 compared with 0 and 3-h treatment groups. CA2, carbonic anhydrase II; CF, cystic fibrosis.

To see if the decrease in CA2 expression is a direct result of CFTR dysfunction, cultured primary WT MNE cells were treated with CFTR Inhibitor-172 for 3, 24, and 48 h (Fig. 4). A representative gel is shown in Fig. 4*A* with quantification normalized to actin expression shown in Fig. 4*B*. Treatment for 24 and 48 h led to a significant decrease in CA2 protein expression suggesting that CFTR serves as a direct link to its expression.

### Treatment with Carbonic Anhydrase II Activator l-Phenylalanine Improves Endosomal Transport and Microtubule Dynamics in CF

**Figure 5.**
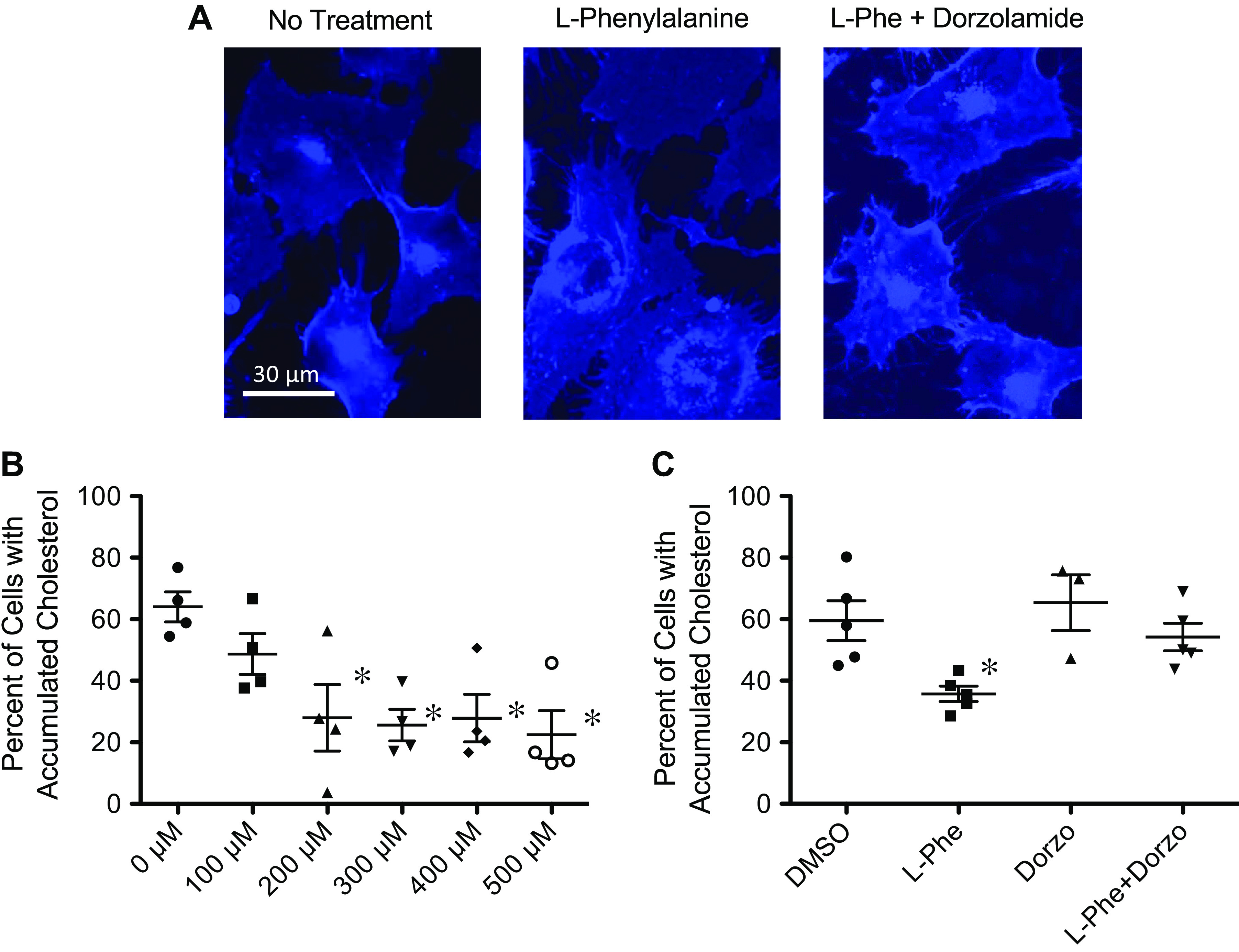
l-Phenylalanine (L-Phe) improves endosomal transport in CF-IB3 cells. IB3 cells were treated for 24 h with vehicle or L-Phe in concentrations ranging from 100 to 500 μM and stained with filipin. *A*: representative samples are portrayed here. *B*: L-Phe reduces cholesterol accumulation in CF-IB3 cells in a dose-dependent fashion. Images were quantified for perinuclear accumulation. Data are presented in graphical form as percentage of cells with perinuclear accumulation (*n* = 4). *C*: improved cholesterol accumulation was blocked by CA2-selective inhibitor, dorzolamide (15 µM) (*n* = 5). Significance determined by ANOVA with Newman–Keuls post hoc test compared with no treatment group; *P < 0.05. CA2, carbonic anhydrase II; CF, cystic fibrosis.

The essential amino acid l-phenylalanine has been shown to activate carbonic anhydrase, and most significantly human CA2, so it is used as a CA activator in this study to further define CA’s role in CF (39, 40). We have previously published that CF cells and tissues exhibit significant perinuclear accumulation of free cholesterol due to impaired endosomal transport along microtubules (4, 8). We use cholesterol trafficking as an indirect marker of intracellular transport efficiency in CF cells and can test the efficacy of interventions on this pathway. S9/IB3 CF model cells were treated with increasing concentrations of l-phenylalanine (L-Phe), 100–500 µM, to determine whether or not activation of CA2 is sufficient to restore this intracellular transport as measured by intracellular cholesterol movement. Cells were stained with filipin and quantified for perinuclear cholesterol accumulation. We found that L-Phe treatment does in fact restore intracellular transport (Fig. 5*A*) in a dose-dependent manner (Fig. 5*B*). We could not detect an increase in overall CA activity with the sensitivity of the assays used in Fig. 1 with L-Phe treatment, therefore we probed for specificity of action biochemically. The effect of L-Phe can be blocked with the CA2-selective inhibitor dorzolamide, as shown in Fig. 5, *A* and *C*, suggesting a specific role for CA2.

**Figure 6.**
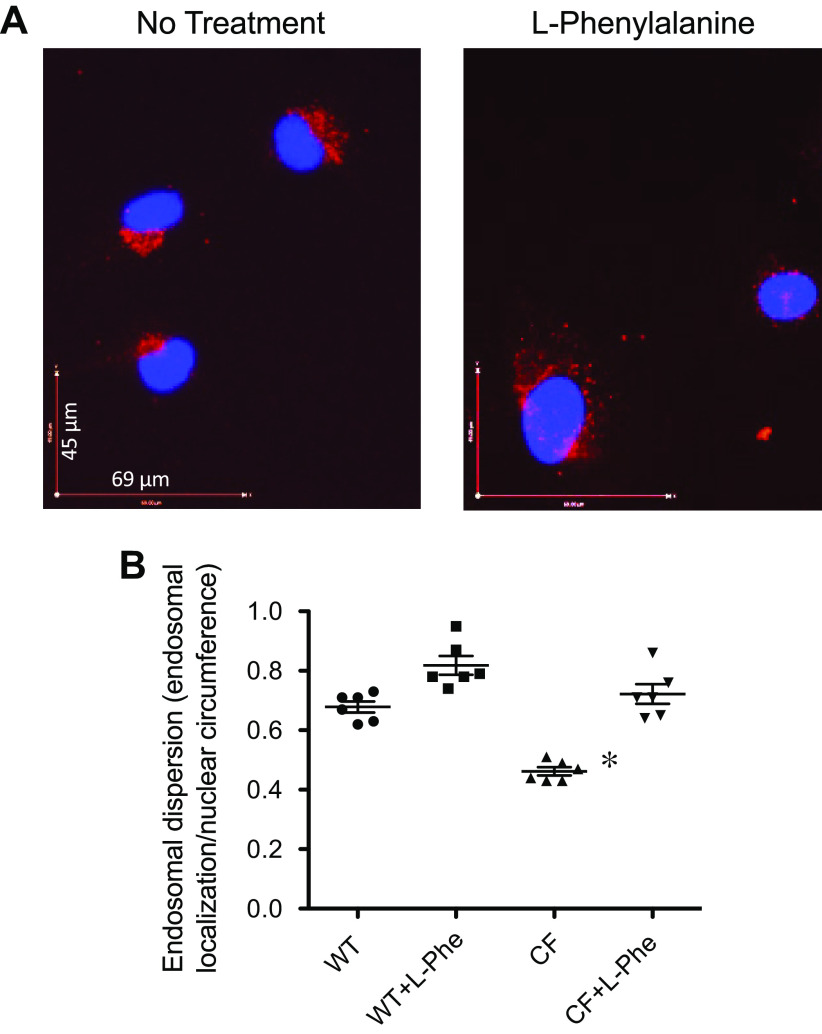
l-Phenylalanine restores late endosomal transport, as demonstrated by redistribution of Rab7 stain around the nucleus and throughout the cytoplasm. *A*: representative images showing Rab7 imaging in IB3 cells treated with vehicle or l-phenylalanine (L-Phe) (500 µM, 24 h). *B*: quantification of Rab7 distribution in cells, reported as endosomal dispersion defined as distribution of Rab7 around a nucleus compared with the circumference of the nucleus as described in methods. Significance determined by ANOVA with Newman–Keuls post hoc test compared with WT no treatment group; *P < 0.05, *n* = 6. WT, wild-type.

To test the efficacy of L-Phe more directly on endosomal transport, we also assessed endosomal transport by immunostaining for the late endosomal marker Rab7 with similar results (Fig. 6, *A* and *B*). These data suggest an effect of L-Phe is modulating endosomal trafficking directly, which suggests an involvement of microtubule regulation as we have shown previously in CF cells.

**Figure 7.**
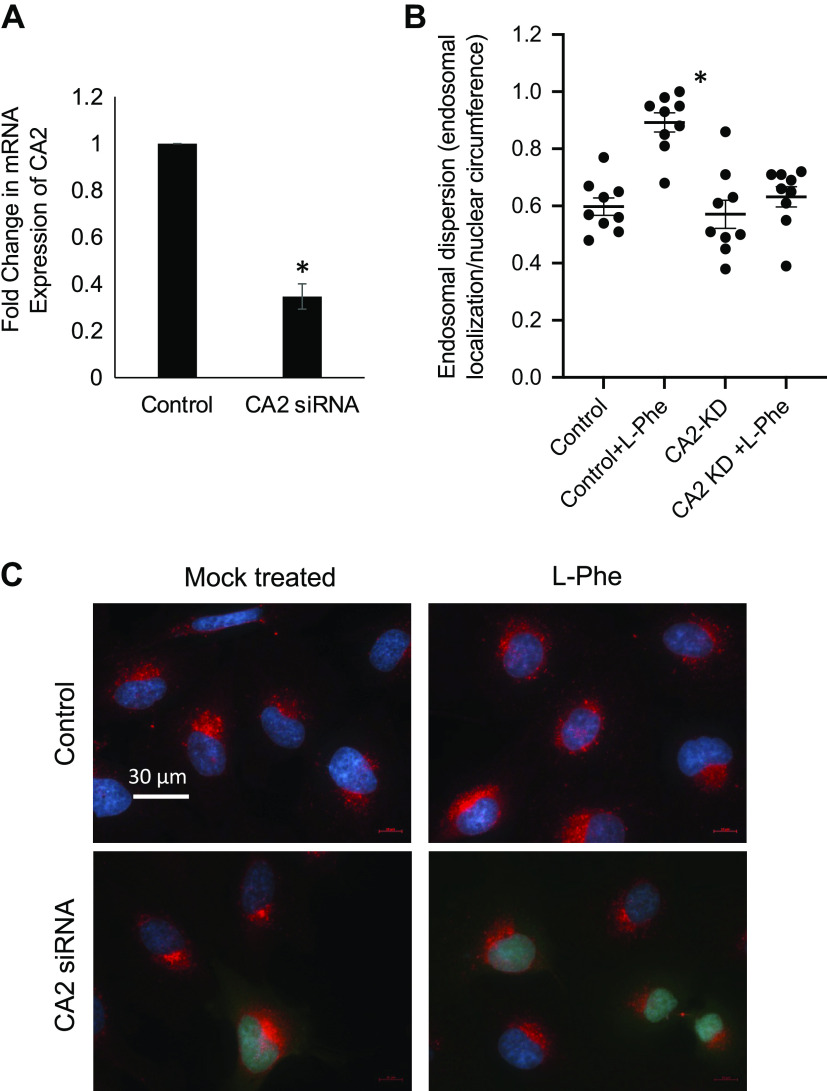
Knockdown of CA2 expression in IB3 cells blunts the impact of L-Phe on endosomal transport in CF IB3 cells. *A*: CA2 mRNA content in IB3 cells treated with control or CA2 siRNA determined by qPCR. Data reported as fold difference relative to control-treated cells (*n* = 3, *P < 0.05). *B*: quantification of Rab7 distribution in cells, reported as endosomal dispersion defined as distribution of Rab7 around a nucleus compared with the circumference of the nucleus as described in methods. Significance determined by ANOVA with Newman–Keuls post hoc test compared with WT no treatment group; *P < 0.001, *n* = 11 for control and *n* = 10 for CA2 siRNA-treated groups. *C*: representative images showing Rab7 imaging in IB3 cells treated with vehicle or l-phenylalanine (L-Phe) (500 µM, 24 h) in the presence of either control siRNA or CA2 siRNA. CA2, carbonic anhydrase II; CF, cystic fibrosis; WT, wild-type.

To confirm that CA2 activation is the mechanism by which L-Phe is reversing endosomal transport, CA2 expression was knocked down by shRNA in IB3 cells and tested for responsiveness to L-Phe. It was predicted that knockdown of CA2 would result in a lack of correction of the endosomal transport phenotype by L-Phe. Since CA2 expression is already low in these cells, knockdown efficiency was determined by qPCR and accessible mRNA levels are reduced by ∼70% by CA2 siRNA (Fig. 7*A*). In cells treated with CA2 siRNA, perinuclear Rab7 staining is present and fails to be corrected by L-Phe, whereas control shRNA-treated cells respond to L-Phe as seen by more redistribution of Rab7 (Fig. 7, *B* and *C*). These data suggest that L-Phe is acting through CA2 even though CA2 levels are already low in this particular CF cell model.

**Figure 8.**
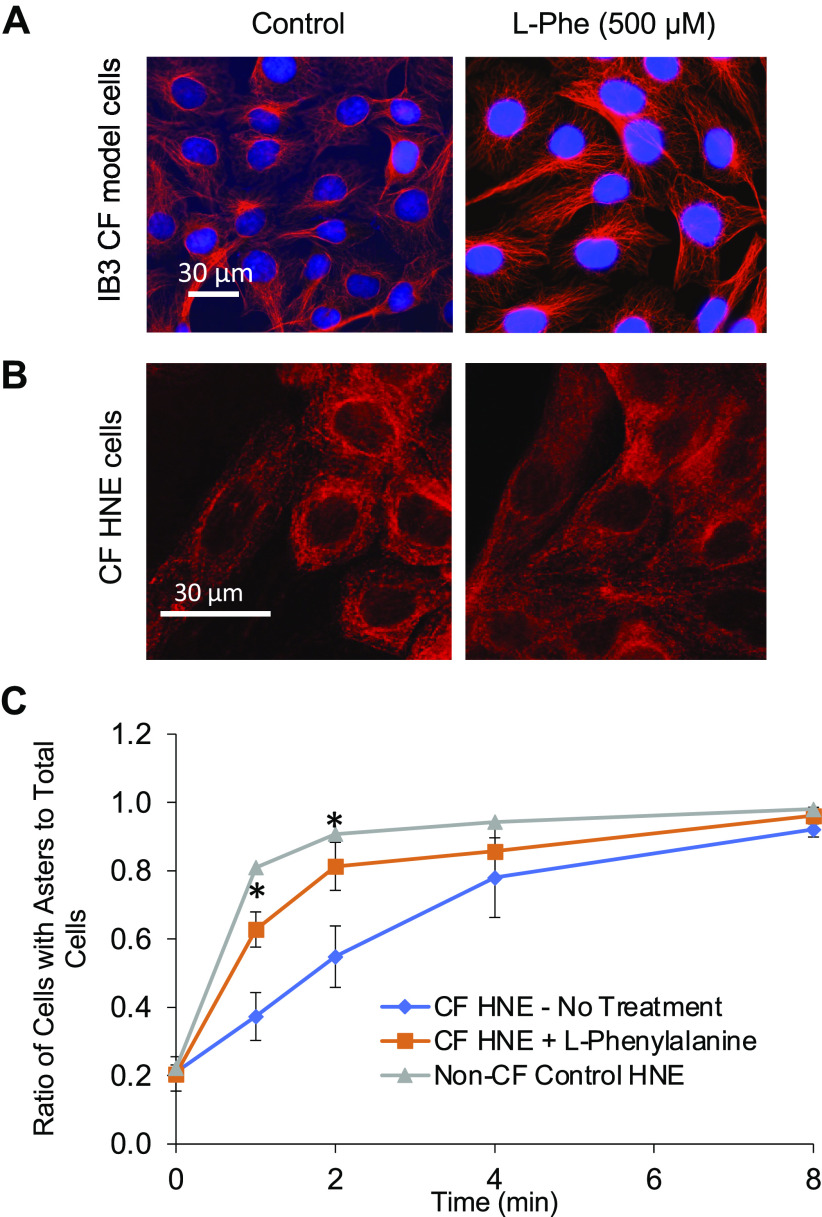
l-Phenylalanine (L-Phe) improves microtubule structure and restores microtubule regulation/elongation in CF cells. *A* and *B*: effect of L-Phe (500 µM, 24 h) on microtubule structure in CF model cells (*n* = 4; *A*) and CF human nasal epithelial (HNE) cells (*n* = 3; *B*). *C*: HNE cells were treated for 24 h with vehicle or L-Phe (500 μM), then depolymerized on ice prior to adding prewarmed media at designated time points. Cells were stained for α-tubulin. Images were quantified by determining the ratio of cells with an aster (signifying microtubule formation) to total cells at the various time points; *n* = 3. Microtubule elongation was more rapid in CF HNE cells treated with L-Phe compared with those that were untreated, as demonstrated by significantly higher ratios of aster formation at 1 and 2 min in treated CF HNE cells. Non-CF microtubule elongation curve is shown for comparison. Significance determined between CF and CF + L-Phe by *t* test at each time point; *P < 0.05. CF, cystic fibrosis.

To further determine if L-Phe restores intracellular transport through an effect on microtubule regulation, we analyzed the effect of treatment on microtubule elongation as we have previously published (2,3, 9). One observation of CF microtubule structure is that they appear less dense and do not extend all of the way to the periphery. We have shown that treatment with ibuprofen restores CF microtubule structure to more WT profiles (3). In Fig. 8, we performed microtubule assays in primary HNE cells, determining the rates of repolymerization in the presence and absence of L-Phe (500 µM). Figure 8, *A* and *B* visually demonstrate the effect of L-Phe treatment on microtubule structure in CF IB3 cells and in primary CF HNE cells, respectively. Figure 8*C* demonstrates that the rate of polymerization is reduced in CF HNE cells consistent with our previous work and restored to WT levels with L-Phe treatment.

### Control of CF Cellular Phenotypes by Soluble Adenylate Cyclase

**Figure 9.**
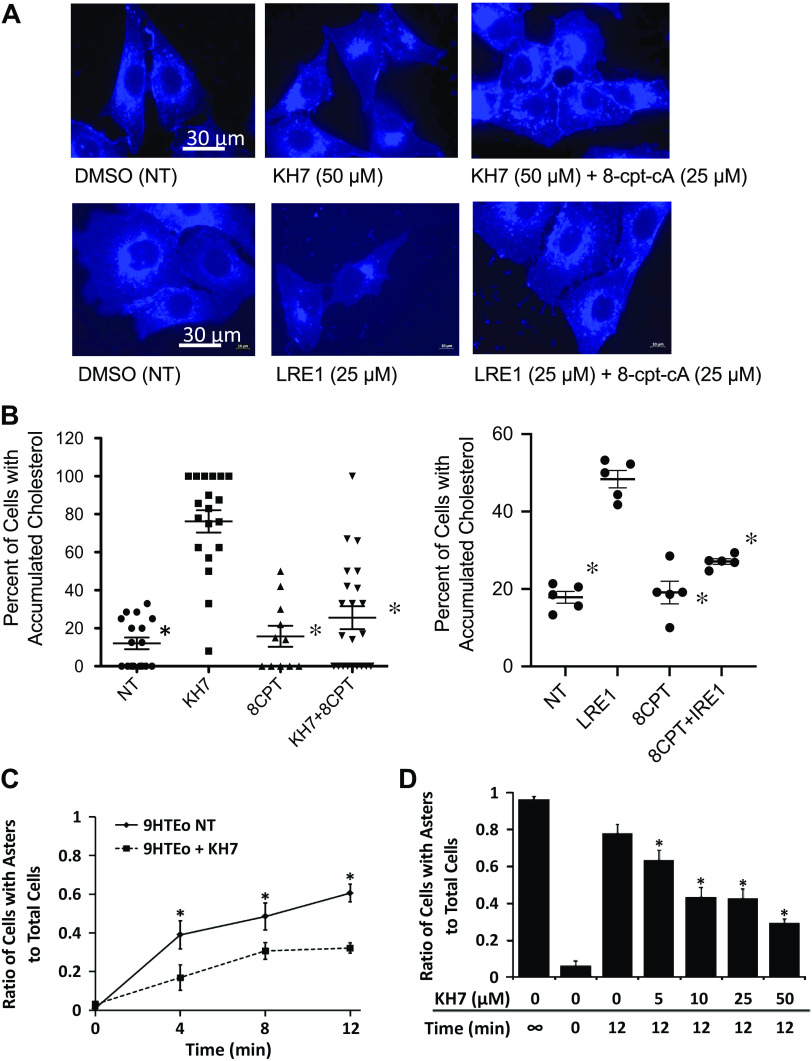
KH7 treatment of 9/HTEo- cells mimics CF cellular phenotypes. *A*: representative images of 50 µM KH7 or LRE1 (25 µM) treatment replication of perinuclear cholesterol accumulation observed in CF cells stained with filipin compared with vehicle group and 25 µM 8-cpt-2-*O*-methyl-cAMP (8-cpt-cA). *B*: quantitative analysis of perinuclear cholesterol accumulation. Each replicate is the average of cells showing perinuclear cholesterol accumulation on a representative area of a coverslip (*left*: *n* = 17 for NT, *n* = 19 for KH7, *n* = 11 for 8CPT, and *n* = 22 for KH7 + 8CPT; *right*: *n* = 5 for NT, *n* = 5 for LRE1, *n* = 5 for *CPT, and *n* = 5 for 8CPT + LRE1). Significance determined by ANOVA with Newman–Keuls multiple comparison test; *P < 0.05 compared with KH7- or LRE1-treated group, respectively. *C*: microtubule elongation over time in vehicle or with KH7 (50 µM) treatment. Significance determined by unpaired *t* test at each time point; *P < 0.05, *n* = 7 for NT, *n* = 5 for KH7. Data are presented as ratio of cells showing asters compared with total cells. *D*: dose dependency of KH7 effect from 0 to 50 µM treatment. Data shown from 12-min point only. Error bars represent SE. Significance determined by ANOVA with Newman–Keuls multiple comparison test; *P < 0.05 compared with 0 µM KH7-treated group, *n* = 5 for each. CF, cystic fibrosis; NT, no treatment.

Given our previous findings that EPAC1 activation is a key step in microtubule regulation in CF cells, we explored whether the HCO_3_^−^-sensitive soluble adenylate cyclase (sAC) was involved in this pathway. Inhibition of sAC with the selective inhibitor KH7 (50 µM) in 9/HTEo- cells leads to CF-like perinuclear cholesterol accumulation (Fig. 9, *A* and *B*), consistent with what we have recently demonstrated (41). To test whether the effects of KH7 were mediated through EPAC1 as we have shown in CF cells, we treated cells with the EPAC1 selective activator 8-cpt-2-O-Me-cAMP (8-cpt-cA) to see if that would overcome the effects of sAC inhibition. Again, consistent with our CF findings, 8-cpt-cA reverses (25 µM) KH7-induced cholesterol accumulation (9). To verify that sAC is the mechanistic target, a second sAC inhibitor, LRE1 (25 µM) was used. Results are identical to those obtained with KH7 supporting the role of sAC in this pathway. It was also tested whether KH7 treatment could replicate microtubule phenotypes and induce slowing of microtubule formation rates (Fig. 9*C*). The effect of KH7 was also found to be dose-dependent (Fig. 9*D**).* These data demonstrate that sAC is a strong candidate as a regulator of intracellular transport through microtubule dynamics influence in CF cells.

### Restoration of Intracellular Transport by CA2 Activation Occurs in a sAC-Dependent Manner

**Figure 10.**
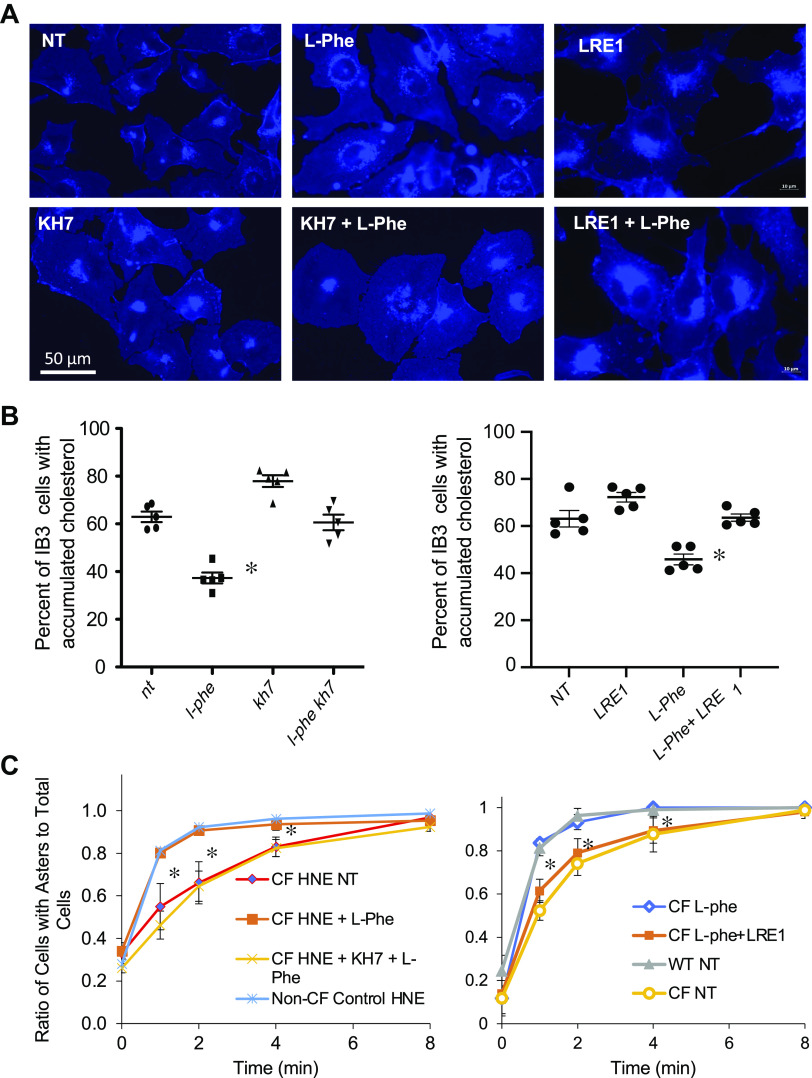
Restoration of intracellular transport and microtubule elongation by l-phenylalanine can be blocked by soluble adenylate cyclase (sAC) inhibition. *A*: representative images of IB3 cells treated for 24 h with vehicle, l-phenylalanine 500 μM, and/or sAC inhibitors KH7 (25 μM) or LRE1 (25 µM) and stained with filipin. *B*: images were quantified for perinuclear cholesterol accumulation; (*n* = 4). Data are presented as percentage of cells with perinuclear accumulation. Significance determined by ANOVA with Newman–Keuls post hoc test compared with no treatment group; *P < 0.05; *left* with KH7, *right* with LRE1 as the sAC inhibitor. *C*: HNE cells were treated for 24 h with vehicle or L-Phe (500 μM) in the presence or absence of sAC inhibitors KH7 (*left*) or LRE1 (*right*) then depolymerized on ice prior to adding prewarmed media at designated time points. Cells were stained for α-tubulin. Images were quantified by determining the ratio of cells with an aster (signifying microtubule formation) to total cells at the various time points; (*n* = 3). Significance determined between CF and CF + L-Phe by *t* test at each time point; *P < 0.05. CF, cystic fibrosis; HNE, human nasal epithelial.

It was demonstrated that L-Phe effects on intracellular transport are sensitive to the CA2 inhibitor dorzolamide. To test the hypothesis that the activation of CA activity leads to sAC activation and subsequent restoration of intracellular transport in CF cells (Fig. 10), the experiments with L-Phe demonstrating improved intracellular transport were repeated in the presence of KH7 or LRE1. Inhibition of sAC with either sAC inhibitor does block the effects of L-Phe, permitting the dysfunctional cellular transport to persist as demonstrated by the increased perinuclear accumulation of cholesterol to CF profiles as shown in Fig. 10*A*. The improved microtubule dynamics observed with L-Phe were also disrupted by the addition of either KH7 or LRE1 to L-Phe in microtubule elongation assays performed in CF and non-CF HNE cells (Fig. 10*B*). These data support the role of sAC activation in the benefits of L-Phe treatment. However, direct examination of CA activity in primary MNE cells treated with L-Phe did not show any increase in activity, suggesting that effects may be subtle and localized. Figure 10*C* demonstrates L-Phe correction of microtubule/aster formation in CF HNE cells and the role of sAC in that correction. L-Phe restores aster formation in CF HNE cells, but the effect is fully blocked by the sAC inhibitors KH7 (*left*) or LRE1 (*right*). Significance between CF untreated (NT) or L-Phe treated is determined at each time point.

### L-Phe Does Not Activate CFTR

Given the possibility that L-Phe could be reversing these cellular effects by activating mutant CFTR, we tested the effect of L-Phe on CFTR function. F508del homozygous HNE cells were treated with L-Phe and then analyzed using the Ussing chamber. As shown in Table 1, L-Phe had no significant effect on CFTR function in primary CF HNE cells.

**Table 1.** Effect of L-Phe on CFTR function in F508del/F508del HNE cells

Treatment	No Treatment (Isc)	L-Phe, 500 µM (Isc)	*P* Value
Baseline	44.6 ± 6.6 µA/cm^2^	41.2 ± 9.2 µA/cm^2^	NS
Amiloride, ΔIsc	0.0 ± 0.1 µA/cm^2^	–0.1 ± 0.1 µA/cm^2^	NS
Forskolin/vx-770, ΔIsc	2.9 ± 0.1 µA/cm^2^	2.7 ± 0.3 µA/cm^2^	NS
Inh172, ΔIsc	1.4 ± 0.1 µA/cm^2^	1.1 ± 0.1 µA/cm^2^	NS

Values are means ± SE. l-Phenylalanine (L-Phe) has no effect on CFTR function in primary CF HNE cells homozygous for the F508del mutation. Cells were treated with 500 µM L-Phe for 24 h. There were no significant differences in resistance values after any treatment. Significance determined by *t* test; *n* = 3. CF, cystic fibrosis; HNE, human nasal epithelial; NS, not significant.

## DISCUSSION

In this study, we have shown that sAC is a key regulator of processes that are altered in CF cells including microtubule dynamics and intracellular transport. We have also demonstrated in both human and mouse primary nasal epithelial cells, as well as CF model cells, that CA2 displays reduced expression. That expression change is directly related to CFTR function as demonstrated by the dose response in MNEs. This relationship may represent a feedback regulatory system since sAC activity has been shown in previous studies to be a significant regulator of CFTR function (42–45). Intracellular transport and microtubule dynamics in CF cells can be reversed to WT profiles by the addition of L-Phe, a reported activator of CA activity. Though this paper demonstrates a CA2-related mechanism, there are multiple CAs and their coordinated impact on CF cell biology still needs to be determined. The manifestations we see in CA2 may be unique to our specific model systems. However, to our knowledge, this study is the first to directly link CA expression to cellular phenotypes in CF and may shed light on how mutations in CA12 are able to clinically mimic many aspects of CF disease. Future work will endeavor to fully characterize the role for both CFTR function and the activity of various CAs on intracellular buffering power, and the activity of other H^+^ and HCO_3_^−^ transporters expressed in MNE and other CF model systems.

Mechanistically, we propose that this study builds on our previously published work demonstrating a distinct role of EPAC1 in microtubule regulation and related cellular phenotypes (9). Specifically, we propose that CA activation results in activation of the bicarbonate-sensitive sAC leading to stimulation of EPAC1 and microtubule regulation. Both pharmacological inhibition and knockdown of CA2 expression support a role for CA2 specifically in this mechanism, at least in this model system. Two separate inhibitors of sAC activity block the effects of L-Phe on microtubule regulation support a role for sAC in the mechanism.

A number of studies to date have suggested that carbonic anhydrases play an essential role in CF; however, none of these investigations have been able to associate CA activity with intracellular functional phenotypes (13, 14, 36). Here, we were able to demonstrate a clear functional link between CFTR and carbonic anhydrase expression, as inhibition of CFTR with inh172 leads to a significant decrease in CA2 protein expression. Overall, RNA and protein expression of cytosolic CA2 is decreased in CF, with some patient-to-patient variability in primary HNE cells.

Our data demonstrate an overall decrease in CA function in CF MNE cells compared with controls. These data do not identify the specific CA or combination of CAs that are altered in CF and may be related to CF pathogenesis. The strongest argument for a role of carbonic anhydrase function being relevant to CF outcomes is Lee et al.’s (36) report of individuals with CA12 mutations presenting with a CF phenotype, including elevated sweat Cl^–^ and lung disease, who were found to have impaired CA12 function. Similarly, support for a potential relationship between CFTR and CA2 can be seen in mouse models of knocked-out anion exchange protein (AE2), another HCO_3_^−^ transporter, which has diminished CA2 expression just as we observe in CF cells (37). Tang et al. (14) demonstrated a normal host response to infection via lymphocytes triggering carbonic anhydrase-dependent HCO_3_^−^ secretion, which was inhibited by knocking out lymphocyte CFTR. This suggests that CFTR may be crucial to promoting epithelial HCO_3_^−^ secretion by increasing CA expression. In addition, treating *Cftr*-deficient mice with rosiglitazone, a synthetic peroxisome proliferator-activated receptor-γ (PPAR-γ) ligand, increased CA2 and CA4 expression leading to increased secretion of HCO_3_^−^ and decreased severity of disease in mice (13). There is also evidence that polyps, frequently found in patients with CF, demonstrate significantly decreased expression of carbonic anhydrase compared with normal nasal mucosa (46). Walker et al. (38), however, show no significant change in CA2 expression in Cftr KO crypt cells, but a significant decrease in CA9 where we show no change in CA9 expression. These expression differences may represent cell type-specific regulation of HCO_3_^−^ transport.

The present study further links disrupted carbonic anhydrase regulation related to CFTR dysfunction to the downstream cell signaling alterations, including impaired intracellular transport and weakened microtubule dynamics, which we have shown are closely tied to inflammation in CF (2, 7). Correction of microtubule defects in CF mice restores growth and weight gain defects, as well as responses to airway bacterial challenge (6, 7). Studies demonstrating that the loss of CA12 function leads to CF-like disease offer compelling evidence that these pathways are key to the pathogenic mechanisms of CF and offer a novel site of intervention that might be exploited to control secondary symptoms of CF. It is not clear if CFTR modulator therapy is able to reverse inflammatory phenotypes and targeting CA function could be an approach to address these symptoms. Further studies on how inflammatory pathways are affected by specific CAs will need to be further addressed.

The effect of L-Phe was demonstrated to be dependent on sAC activity. Either the effect of L-Phe on CA activity is small but very localized to mediate the sAC-dependent changes in microtubule dynamics and intracellular transport or L-Phe is acting directly on sAC. Future studies again will focus on specific CAs and how L-Phe modulates their function in relation to intracellular transport and related sequelae.

## GRANTS

This work is supported by Cystic Fibrosis Foundation (CFF) Grant KELLEY17G0, a CFF fellowship award (to K.B.), and CFF Grant RDP R447-CR11 and NIH Grant DK113197 (to W.F.B.). K.B. was also supported by NIH Grant 5T32HL125245.

## DISCLOSURES

No conflicts of interest, financial or otherwise, are declared by the authors.

## AUTHOR CONTRIBUTIONS

K.B., F.J.M., and T.J.K. conceived and designed research; K.B., D.A.C., P.Z., B.L., and F.J.M. performed experiments; K.B., B.L., W.F.B., F.J.M., and T.J.K. analyzed data; K.B., W.F.B., and F.J.M. interpreted results of experiments; K.B., F.J.M., and T.J.K. prepared figures; K.B. drafted manuscript; K.B., D.A.C., F.J.M., and T.J.K. edited and revised manuscript; K.B., D.A.C., P.Z., B.L., W.F.B., F.J.M., and T.J.K. approved final version of manuscript.
